# Management of severe Stevens–Johnson syndrome

**DOI:** 10.1186/1471-2334-13-S1-P95

**Published:** 2013-12-16

**Authors:** Dan Calota, Silvia Condrea, Simona Nițescu, Cristina Huian, Natalia Marcenco, Ileana Boiangiu, Gheorghiță Jugulete

**Affiliations:** 1 Emergency Clinical Hospital for Plastic, Reconstructive Surgery and Burns, Bucharest, Romania; 2 National Institute for Infectious Diseases “Prof. Dr. Matei Balş”, Bucharest, Romania; 3 Carol Davila University of Medicine and Pharmacy, Bucharest, Romania

## Background

Severe exfoliative diseases of the skin (Erythema multiforme, Stevens–Johnson syndrome - SJS, toxic epidermal necrolysis -TEN) are a result of an immunological reaction to foreign antigens, most frequently caused by drug consumption. It is characterized by extensive damage to the skin and mucous membranes due to detachment at the dermoepidermal junction, very similar to post-combustional lesions.

This article wants to emphasize on the role of the plastic surgeon in the management of exfoliative diseases of the skin and the reason why patients suffering from SJS or TEN should be directed and treated in burn intensive care units, where they can receive adequate fluid resuscitation, nutritional support, wound care and physical therapy.

## Methods

We have carried out a retrospective study between September 2012 and April 2013 on all patients with SJS or TEN treated in our Burn ICU based on medical records, clinical examination, local examination, dynamic evaluation of vital parameters (blood, wound secretion, vital functions).

## Results

All patients presented local or systemic complications that were treated adequately. Excepting one patient who died due to sepsis, all other patients survived.

## Conclusion

Treatment of severe exfoliative skin diseases is a complex process that requires a multidisciplinary approach, but the most important decision is to direct the patient to a burn intensive care unit.

